# The History of and Advances in Newborn Screening: Where Do We Stand?

**DOI:** 10.3390/genes17030359

**Published:** 2026-03-23

**Authors:** Sharon Anderson, Milen Velinov

**Affiliations:** 1 Division of Advanced Nursing Practice, Rutgers School of Nursing, Newark, NJ 07101, USA; sharon.anderson@rutgers.edu; 2 Department of Pediatrics, Rutgers Robert Wood Johnson Medical School, Child Health Institute of New Jersey, New Brunswick, NJ 08901, USA

**Keywords:** newborn screening, genome sequencing, genetic disorders

## Abstract

To comprehend the current state and future of newborn screening (NBS), it is essential to understand its history. Over the past six decades, this well-established and exemplary population-based screening program has been guided by screening principles dating back more than half a century. Advances in laboratory and point-of-care testing, diagnostic methods, and a surge of available treatments and even cures have made it challenging to balance screening criteria that have not kept pace with the current landscape. The availability to screen as well as the demand from parents and stakeholders to screen for more and increasingly complex conditions while limiting the retention of NBS specimens and genetic material has been both exciting and challenging. This paper shares the history of NBS in the United States, followed by the development and integration of genomic sequencing as a complement to current practice. It explores evidence supporting the concomitant use of biomarker- and DNA-sequencing-based approaches for NBS, how disorders are selected for inclusion, and available treatments, and offers recommendations regarding what to consider and how to proceed in this ever-changing NBS landscape.

## 1. Introduction

As of November 2025, the Online Mendelian Inheritance of Man (OMIM) database listed 7664 single-gene disease phenotypes associated with 5034 single genes. Since 2014, approximately 300 newly discovered, primarily single-gene disorders have been added to the OMIM database annually [1]. Approximately 70% of all single-gene disorders have a childhood onset, and timely diagnosis and management, that may improve long-term clinical outcomes if started early, is available for more than 500 genetic disorders.

Screening of newborns for treatable, early-onset disorders has been implemented in the United States (U.S.) and abroad for more than 60 years, and evidence suggests that early diagnosis and treatment improve long-term outcomes and reduce costs. Based on a 2020 Centers for Disease Control and Prevention report, between 2015 and 2017, 34 newborns with such disorders were identified in the U.S. for every 10,000 screened infants [2]. As of December 2025, the U.S. Recommended Uniform Screening panel (RUSP) included 40 core (recommended) conditions and 26 secondary conditions. Depending on each state’s capacity to screen all newborns, the types and number of conditions screened vary across states. In addition to the lack of standardization across U.S. states, current recommendations fail to capture all conditions that could be included. Even in the most comprehensive programs, only 10–25% of the conditions that meet screening eligibility recommendations, including available treatments, are included in routine state-based programs.

Another limitation of the current system is the high number of false-positive results. The development of advanced technologies for rapid genome sequencing (GS), however, provides an opportunity to significantly expand the number of disorders screened and reduce false positives. Beginning with exome and now genome next-generation sequencing, several international genomic sequencing pilot and research projects to support rare disease detection in newborns were underway in the U.S. and abroad (Table 1). While these advanced approaches and research create opportunities for the inclusion of an unlimited number of screened disorders, unresolved issues remain regarding which specific disorders to include in such a program, which DNA variants are candidates for NBS based on variable penetrance and diagnostic and treatment timeliness, and how to build the infrastructure to manage newly diagnosed rare genetic conditions promptly.

## 2. The History of Newborn Screening

Newborn screening is an exemplary public health program that aims to detect, on a population scale, health conditions that, when diagnosed and treated early, reduce infant and early childhood morbidity and mortality. Newborn screening began in the early 1960s when Robert Guthrie, M.D., Ph.D., developed a screening assay for phenylketonuria using filter-paper dried blood spots [14]. Although expansion was slow between the early 1960s and the 2000s, the introduction of tandem mass spectrometry (MS/MS)-based multiplex testing in the early 2000s triggered exponential growth. Currently, NBS methods can screen for and, in some cases, diagnose a range of disorders, including amino acid, fatty acid, organic acid, endocrine, hemoglobin, lysosomal storage, and other conditions.

### 2.1. The Screening Criteria

Formulated by Wilson and Jungner in 1968, the ten principles of population-based screening remain broadly relevant and have guided decisions regarding disease inclusion in NBS programs for the last half century [15]. Not criteria, per se, but considerations to be worked through, these screening principles state (1) the condition should be an important health problem, (2) there should be an accepted treatment for patients with recognized disease, (3) facilities for diagnosis and treatment should be available, (4) there should be a recognizable latent or early symptomatic stage, (5) there should be a suitable test or examination, (6) the test should be acceptable to the population, (7) the natural history of the condition including development from latent to declared disease, should be adequately understood, (8) there should be an agreed upon policy on whom to treat, (9) the cost of the case-finding should be economically balanced in relation to possible expenditure on medical care as a whole, and (10) case finding should be continuing process and not a “once and for all” process [15]. While these principles were strong and applicable to NBS, they focused on adult screening programs, not on the introduction of NBS at that time.

Wilson and Jungner principles have been the gold standard and guided decisions regarding condition inclusion to NBS for the past 50 years, but newer and expanding technologies, including genomic-based methodologies and the changing scientific and societal views on the ethical, legal, and social implications of population-based screening practices, raise concerns surrounding the relevance of these criteria. Over the last two decades, these principles have been reviewed, and alternative and augmented principles have been presented.

Despite decades of uniform reference to the Wilson–Jungner principles, screening, inclusion, and selection criteria for NBS remain highly variable. Furthermore, as we learn more about genetic diseases, we recognize that the natural history and phenotypic diversity of rare diseases known in 1968 are far different from what is known today (and are still incompletely understood). As such, several authors have suggested incorporating current principles that incorporate modern medical technologies and perspectives, as well as the ethical, legal, and societal perspectives of patients, stakeholders, and the public [16].

For example, in 2008, Andermann et al. [17] proposed screening criteria based on recognized need. They recommended screening objectives and a well-defined evaluation plan for a target population based on scientific evidence of screening effectiveness. They recommended integrating education, testing, clinical services, and program management within the system and aligning quality assurance to mitigate screening risks. They recommended informed choice, confidentiality, respect for autonomy, and equitable access to screening for the entire target population. Overall, the benefits of screening should outweigh the harm [17].

In 2018, after a comprehensive systematic review and a modified Delphi consensus process, a refined set of consolidated principles was developed. Principles proposed by Dobrow et al. [18] are categorized into three domains: (a) disease/condition principles, (b) test/intervention principles, and (c) program/system principles. Disease/condition principles include understanding the epidemiology and natural history of the disease or condition, including a pre-clinical phase, and identifying the target population (e.g., age). Regarding test/intervention principles, the screening test should be appropriate, accurate, reliable, and reproducible; acceptable and affordable to the target population; and safe to perform. Results should have well-defined, agreed-upon reference ranges, and the course of action for individuals who screen positive should include diagnostic testing, intervention, and follow-up care tailored to the disease’s natural history. Finally, screening program infrastructure, including but not limited to financial support and accessibility to information, scientific technology, and equipment, is essential to a successful screening program. Screening must be well-coordinated and integrated into the broader health system. In addition to being clinically sound, screening should be socially and ethically acceptable to those screened, clinicians, and society, with the benefits outweighing potential harm. Finally, the program should ensure ongoing quality control and ensure that all aspects are cost-effective [18].

After reviewing the literature, Schnabel-Besson et al. [16] categorized the 10 principles of Wilson and Jungner to propose four criteria for future NBS as follows: clinical (natural history of the disease and course), diagnostic (acceptable and suitable test), therapeutic (available and accessibility to treatment focusing on cost of case-finding and medical expenditures), and program management, incorporating ethical, legal and social implications of NBS and programs. They highlight the importance of evidence-based practice, informed choice, policymaking, cost-effectiveness, and quality assurance when revising screening criteria [16].

### 2.2. Newborn Screening in the United States

Mandatory in the U.S., the District of Columbia, Guam, and Puerto Rico, NBS is state-based and often driven by population demographics and resource allocation. To help standardize the expansion screening across and between states, the Advisory Committee on Heritable Disorders in Newborns and Children (ACHDNC, formerly known as the Secretary’s ACHDNC and Discretionary ACHDNC) was established in 2005. Authorized and funded in 2008, the Newborn Screening Saves Lives Act of 2007 [7] provides federal support for newborn screening programs and charged the ACHDNC with developing a framework for national screening. Comprising individuals with diverse expertise and perspectives, this science-driven federal advisory committee deliberated on conditions nominated for screening and advised the Secretary of the Department of Health and Human Services (the Secretary).

Based on ethical and public health principles, such as the Wilson and Jungner screening principles [15], this committee reviewed conditions nominated for inclusion and approved or rejected them based on current knowledge about the condition, presentation at a young age, available treatment options, and the ability for the condition to be detected by reliable NBS methodologies. In addition, ACHDNC developed a model decision matrix and explored ways for states to screen for the recommended conditions [19]. Committee recommendations were shared with the secretary, who, in turn, determined which conditions should be included in the Recommended Uniform Screening Panel (RUSP).

Following the dissolution of ACHDNC in 2025, the American College of Medical Genetics and Genomics (ACMG) announced it will establish the ACMG Newborn Screening Coalition to advise the public and NBS programs, develop and conduct evidence-informed reviews, disseminate those reviews and recommendations, and maintain national collaboration, oversight, and evaluation of the RUSP. As much of this work originated with ACMG, it is without question that this important work will continue [20].

### 2.3. Current United States Newborn Screening Recommendations

NBS programs employ a range of biochemical, PCR-based, DNA/next-generation sequencing, and point-of-care methods to test newborns for more than 60 conditions [21]. As of July 2024, the RUSP included 38 core (should be included in every NBS program) and 28 secondary (detected in the differential diagnosis of a core disorder) disorders. At the writing of this manuscript, the majority of the 56 U.S. NBS programs screened for 32 to 38 of the recommended disorders [22]. Given the comparison of screened conditions across NBS programs and the resulting confusion, ACHDNC recommended updates and standardization of disorder names and categories. National work surrounding standardized nomenclature, secondary conditions, and condition-counting frameworks is currently underway through the Newborn Screening Technical Assistance and Evaluation Program (NewSTEPS) of the Association of Public Health Laboratories [22]. While progress is being made, the uniform adoption of standardized nomenclature, including details about the diagnosis of infantile versus later-onset conditions or the specific disorder type (*GALT* deficiency and/or non-classic galactosemia), remains inconsistent, and, as a result, relying solely on the number of conditions screened to compare NBS programs can be problematic.

Although not yet universally screened, newer core disorders include mucopolysaccharidosis Type II (MPS II) as of 2022, guanidinoacetate methyltransferase deficiency (GAMT) as of 2023, and infantile Krabbe disease as of 2024 [18]. Having completed the evidence review, ACHDNC, before issuing its recommendation to include Duchenne muscular dystrophy and early-onset metachromatic leukodystrophy in the RUSP, was dismantled. As such, the ACMG Newborn Screening Coalition finalized recommendations to the Secretary, who included these conditions in the RUSP in December 2025 [23], increasing the number of core disorders on the RUSP to 40 [24].

### 2.4. Newborn Screening Challenges

While the benefits of NBS are well-established, its expansion is not without controversy, and there are opportunities for improvement and areas to explore. Challenges include, but are not limited to, equity, education, and test results, and the economic, quality-of-life, and lifelong costs of care.

A. Screening and Care Equity

With nationally established guidelines, NBS should ensure equity. Although well-intended, there are screening inequities. Due to the limited race/ethnicities in current reference databases, this presents genetic inequities for minority populations. Another consideration is access to specialist care and treatments, which depends on insurance coverage and/or the ability to afford care, and varies by individuals, states, and insurance plans. Furthermore, consideration must also be given to access to care, the diagnosis, and treatment of family members, by proxy.

B. Education

Despite access to patient education through technological platforms, parents often retain little information about NBS provided in the prenatal and postnatal settings, and parents and providers are often confused about the test results, the urgency of that result, and the disorders screened. As such, when implementing NBS changes, lay and professional stakeholders must be educated and informed to ensure the requisite care is provided. Parents must be provided with timely information based on the stage of diagnosis (presumptive versus confirmed) and learning and language preferences. Education also incorporates closing knowledge gaps regarding data and privacy protections.

C. False Positive Rate

Despite improvements in parallel or reflex biochemical and NGS testing, such as phenylalanine and tyrosine ratios for phenylketonuria, succinylacetone for tyrosinemia type 1, and common *CFTR* variant panels for infants with an elevated immunoreactive trypsinogen, NBS false-positive rates remain high. While only a percentage of metabolic NBS referrals across our state, Table 2 shows the number of abnormal NBS referrals to our metabolic service program at Rutgers Health, Robert Wood Johnson Medical School over 10 years. The table shows a high proportion of false-positive referrals. Although not unique to our center, only 8.5% of NBS referrals were confirmed as positive. While evidence is mixed regarding the stress and emotional and mental health sequelae, and, if so, the severity of those effects surrounding false-positive results for parents [25], high false-positive screening rates reduce the potential cost–benefit of NBS programs and the cost-effectiveness of care.

D. Cost of Care

In addition to reducing disease-associated morbidity and mortality, identifying newborns via NBS can reduce the cost of care for many patients with early-onset disorders. While significant cost savings are estimated for the specific disorders included [26,27,28,29,30], conducting high-quality studies to estimate the cost-effectiveness of NBS poses challenges [31].

E. Limited number of included disorders

Perhaps the most significant deficiency in the current NBS is the exclusion of multiple early-onset genetic disorders with existing treatments because there is no available, reliable, and low-cost biomarker screening protocol. As previously stated, more than 500 genetic disorders have available management that may improve long-term clinical outcomes if started early, and approximately 30 to 50 newly discovered single-gene disorders are added to the OMIM database monthly [32]. As such, current NBS research is exploring integrating next-generation GS and artificial intelligence into NBS, while considering the scientific, legal, ethical, and societal implications of this shift.

## 3. Advanced Newborn Screening

During the first decade of the 21st century, advances in next-generation DNA sequencing technology enabled dramatic increases in speed and throughput for sequencing and analyzing large amounts of DNA [33]. As previously discussed, NBS principles and criteria for inclusion are being questioned and have not kept pace with the ever-changing technological methods in GS and rare disease treatment options.

### 3.1. Development of Genomic Sequencing

In the past decade, clinical exome sequencing (ES) and now, GS, have become routine tests in medicine. Rapid and even ultra-rapid GS is currently being established as the standard of care for young children with suspected genetic etiology who are acutely ill [34], with results available within 3 to 5 days. Technological advances and the rapid reduction in the time required to analyze GS data have prompted interest in its application to NBS [35].

As previously noted, several pilot and research studies are exploring the feasibility of next-generation sequencing-based NBS both in the U.S. and abroad [3,4,5,6,7,8,9]. When designing these projects, one consideration is whether to use whole-GS and select genes for the NBS protocol, or to sequence only a panel of selected genes. While panel testing is typically faster to analyze and lower in cost, GS offers the ability to add new conditions to the analyzed gene panel at no additional cost, making it more efficient given the rapid development of new treatment protocols for rare disorders. Selection of conditions to include in screening panels, pre-test counseling and informed consent, and reporting of carriers and variants of uncertain significance are considerations when such protocols are implemented.

### 3.2. The Selection of Disorders for Inclusion

Gene panel selection is key to integrating GS into NBS. Given the multiple aspects to be considered, all included genes should meet the current NBS selection criteria [15,16,17,18,19,20,21,22,23,24]. Because GS provides such a universal assay, all requirements should be met, except for the availability of a low-cost, high-capacity test protocol. When selecting disorders for inclusion, several key factors must be considered. These include, but are not limited to, treatment availability and effectiveness, disease penetrance, and age of onset.

#### 3.2.1. Available Treatments

While there are available treatments for genetic disorders, most are not curative. Although there is limited long-term outcome data, in general, even gene therapy protocols that deliver the missing gene do not provide a complete cure. As such, it is reasonable to question whether the available treatment offers significant improvement in the patient’s quality of life when implemented early in the disease course.

When evaluating the inclusion of genes and gene panels in NBS, there are three main types of disorders:

A. Disorders for which treatment is curative.

A treatment that falls into this category is the FDA-approved ex vivo gene therapy for severe cases of beta hemoglobinopathies [36]. Combined with autologous hematopoietic stem cell transplantation, gene therapy by gene addition and gene editing is a life-changing breakthrough in these inherited single-gene disorders.

B. Disorders for which there is an efficient targeted treatment that slows the disease progression and improves the quality of life.

Examples of NBS disorders identified from routine biochemical screening include metabolic disorders for which early dietary changes help prevent significant morbidity and may even avoid all clinical manifestations, such as phenylketonuria and medium-chain acyl-CoA dehydrogenase (MCAD) deficiency [37,38]. Although not included in the RUSP core disorders, ornithine transcarboxylase deficiency is an inborn error of metabolism with a severe early-onset presentation that requires prompt treatment in most cases [39,40], and Menkes disease, for which copper histidine supplementation is a recently FDA-approved treatment [41,42]. Non-metabolic disorders that fall within this group include spinal muscular atrophy and Duchenne muscular dystrophy, for which gene therapy protocols are available and have been shown to slow the disease progression [43,44].

C. Disorders for which there are no FDA-approved targeted treatments, but for which early implemented non-disease-specific treatments show benefit.

The large group of neurodevelopmental disorders meets these criteria and serves as a helpful example in this context [45]. Evidence supports the idea that early intervention, such as applied behavior analysis, speech, occupational, and physical therapies, as well as developmental interventions, family support, counseling, and training, improves long-term outcomes for neurodiverse individuals.

#### 3.2.2. Penetrance

While phenotypes may vary based on expressivity, GS for conditions with complete or very high penetrance is straightforward. Determining whether and, if so, which conditions with incomplete or reduced penetrance should be included in NBS gene panels is difficult. One example is long QT syndrome. Experts in these conduction disorders recommend prophylaxis for individuals with pathogenic variants who have a normal electrocardiogram [46]. Therefore, inclusion of such a condition is justified despite the reduced penetrance. It seems that penetrance should be considered individually for each condition under consideration for screening.

#### 3.2.3. Age of Onset

Most experts agree that adult-onset conditions should not be included in NBS programs. However, there is no consensus on the age cutoff for inclusion, and some conditions, whether added to the RUSP, have been incorporated into NBS programs.

For example, while males with childhood cerebral X-linked adrenoleukodystrophy are the primary target for NBS, males and females are screened for this. Some affected females, and even some males, may develop later-onset adrenomyeloneuropathy, adrenocortical insufficiency, or remain asymptomatic. Another example is Fabry disease, an X-linked disorder that typically presents in adolescent male patients. Despite symptom development beyond the newborn period or even early childhood, this condition is included in some biomarker-based NBS programs. The rationale behind screening is that, although rare, manifestations may be present as early as age 4 [47].

### 3.3. Pre-Test Counseling

Currently, pre-test genetic counseling and informed consent are completed for GS NBS pilot research studies. While best practice, offering full genetic counseling sessions to all families in clinical NBS programs may not be feasible. Given the complexity of GS-based testing, implementation would likely require providing pre-test information, including electronic links to detailed information and/or applications, the importance and purpose of GS NBS, and a list of the included conditions, available treatments, and information surrounding confidentiality, genetic privacy, and discrimination.

### 3.4. Reporting Carriers and Variants of Uncertain Significance

While carrier status is actionable and may guide future family planning, given the large number of individuals who would require post-test counseling, reporting all carriers and variants of uncertain significance (VUS) would overwhelm any NBS program. The depth and breadth of sharing these results is not feasible and cannot be considered at this time. Since some VUSs would inevitably be reclassified as pathogenic or likely pathogenic in future analyses, it is important to note that failing to report VUSs will lead to false-negative results (missed affected newborns). As with all genetic testing, GS-based NBS has benefits and limitations, and weighing the risks and harms of under- and over-reporting is imperative to current and future program and system success.

## 4. Summary and Recommendations

Based on our long-term experience with biomarker-based NBS and our recent experience with GS-based pilot testing, we suggest the following

1. Combined concomitant implementation of biomarker-based and GS-based NBS with high coordination between the two strategies may be the most efficient approach. GS-based testing may significantly reduce false-positive test results in current biomarker testing. In return, biomarker testing would help diagnose affected newborns with VUSs that GS-based testing would not report. There is an argument that carrying both methods simultaneously would make the screening too costly. This increased cost may be at least partially mitigated by eliminating false-positive biomarker tests, thereby reducing overall costs by avoiding unnecessary follow-up visits and testing for these newborns. For disorders included in the GS-based panel but not in the biomarker panel, some affected newborns with pathogenic variants that, at the time of the report, were considered VUS would be unavoidably missed. Therefore, careful documentation of patients with false-negative results (later diagnosed) would be essential.
2. For the selection of disorders to be included in the GS-based screening, disorder types outlined in the above-formulated treatment groups should include type A (for which treatment is curative) and type B (for which there is an effective targeted treatment that slows disease progression and improves quality of life). Type C disorders (for which there is no FDA-approved targeted treatment, but for which early implementation of non-disease-specific treatments shows benefit) should be evaluated individually to determine the benefits and outcomes of early treatment. The disorders with lower penetrance should also be assessed individually, with the decision based not only on penetrance but also on the benefit of prophylaxis/monitoring for asymptomatic individuals.
3. A comprehensive support system consisting of online information, consenting protocols, and follow-up for positive results is essential.
4. Appropriate insurance coverage for treatments of all included disorders should be mandated for all insurance plans. Concerns regarding access to early intervention programs for newborns diagnosed with neurodevelopmental conditions were previously reported in relation to one of the pilot NBS trials [48]. Universal insurance coverage to address abnormal NBS results would eliminate the possibility of discrimination and increase the program’s cost efficiency.
5. A robust system for short- and long-term follow-up of newborns with abnormal results is essential. Disorders included in GS-based NBS may be sporadic, with true incidence rates unknown, and limited management and outcome data. Information regarding treatment centers and guidelines for all included disorders must be available for all primary care providers.
6. Finally, as we gain knowledge and experience with this advanced technology, it is imperative that the principles and criteria for GC NBS remain fluid. We must continue to assess the benefits and limitations of what has and has not been successful, remain open to adjustments, and be willing to change course based on the best interests and needs of our patients, families, communities, and systems.

## 5. Conclusions

Learning from history and current research and evidence, incorporating GS into current biomarker screening, this expansion should be implemented with consideration of the potential legal, ethical, and societal implications of this screening and in close collaboration between the two methods, with real-time exchange of information on positive results. Ideally, the population must accept this new method, along with the challenges surrounding informed consent, the disorders to be screened for, and the results. Once accepted, the two methods should be run by the same laboratory group. The evidence to date shows program expansion may mitigate existing health care inequalities in diagnosing rare disorders due to unequal access, insurance coverage, and delayed diagnoses among underserved populations. Furthermore, it may lower the long-term cost of care for rare disorders by reducing the costs of intensive care admissions and long-term care for disabled patients, and by improving family planning for affected families. Advanced NBS will help obtain precise population prevalence estimates for various rare disorders and inform management plans accordingly. Most importantly, such an approach will improve the health of the general population.

## Figures and Tables

**Table 1:** Major Genomic Sequencing Newborn Screening Research Initiatives by Country.

Research Project	Participating Country(ies)	Sequencing Modality	Brief Description	Select Citation(s)
BabySeq2	United States	WGS	An extension of the BabySeq Project that applied WES, this project explored the use of genomic sequencing in the newborn period to screen healthy infants from ethnically and racially diverse communities as part of routine pediatric care, to assess current and future health risks.	[3]
BEACONS (Building Evidence and Collaboration for GenOmics in Nationwide Newborn Screening)	United States	WGS	National Institutes of Health-funded initiative to screen up to 30,000 newborns across 10 states for 746 genes linked to 777 conditions.	[4]
BeginNGS	United States	rWGS	Evaluates early detection of actionable childhood-onset disorders using rapid genome sequencing.	[5,6]
GUARDIAN (Genomic Uniform-screening Against Rare Diseases In All Newborns)	United States (New York)	WGS	Research sponsored by Columbia University in partnership with New York-Presbyterian, and the New York State Department of Health, using WGS to screen 100,000 newborns for more than 250 genetic conditions not currently included in standard newborn screening	[7]
BabyDetect	Belgium	Targeted NGS	Research program expanding screening beyond traditional biochemical panels to include genomic conditions.	[8]
BabyScreen+	Australia	WGS	Pilot assessing the feasibility of screening for treatable genetic disorders using genomic sequencing.	[9]
The China Neonatal Genomes Project	China	WGS	National initiative to sequence newborn genomes for early rare disease detection.	[10]
Newborn Genomes Program (Generation Study)	England	WGS	National initiative evaluating the feasibility of integrating WGS into routine newborn screening.	[11]
PERIGENOMED	France	WGS	National pilot evaluating genomic sequencing for rare disease detection in newborns.	[12]
Screen4Care	Italy, France, Germany, the Netherlands, Denmark, Belgium, Spain, and others	NGS/WGS	Multinational initiative aimed at early identification of rare diseases through genomic newborn screening.	[13]

Note: WES = whole exome sequencing; WGS = whole genome sequencing; rWGS = rapid whole genome sequencing; NGS = next generation sequencing.

**Table 2:** Referrals to our NBS Center 2016 through 2025 (10 years).

	Total Referrals	Positive (Confirmed Diagnosis)	VUS/Following Disorder (Number)	Declined Testing Disorder (Number)	Lost to Follow-Up Abnormality (Number)	Expired Before Referral/Confirmatory Testing
Number	349	30	3	2	12	2
Comments			X-ALD VUS (male 1) Pathogenic variant (female 2)	Gaucher disease (2)	GA1 (1)	
					*GALT* (3)	
					Fabry disease (1)	
					Krabbe disease (father, known carrier 30 kb deletion) (1)	
					MPS 1 (2)	
					PA/MMA (2)	
					PA/MMA and GA1 (same baby) (1)	
					Tyrosinemia (1)	
% of Total Referrals to our Program		8.5%	0.85%	0.57%	3.4%	0.57%

Note: X-ALD = X-linked adrenoleukodystrophy; VUS = variant of uncertain significance; GA1 = Glutaric acidemia type 1; *GALT* = GALT-deficiency galactosemia; MPS 1 = mucopolysaccharidosis type 1; PA/MMA = propionic/methylmalonic acidemia.

## Data Availability

The original contributions presented in this study are included in the article. Further inquiries can be directed to the corresponding author.
